# Circulating Cell-Free DNA as an Epigenetic Biomarker for Early Diabetic Retinopathy: A Narrative Review

**DOI:** 10.3390/diagnostics15091161

**Published:** 2025-05-02

**Authors:** Boaz Li, Megan M. Yim, Yu Xuan Jin, Brendan K. Tao, Jim S. Xie, Michael Balas, Haaris Khan, Wai-Ching Lam, Peng Yan, Eduardo V. Navajas

**Affiliations:** 1 Faculty of Medicine, The University of British Columbia, Vancouver, BC V6T 1Z3, Canada; bli1025@student.ubc.ca (B.L.); myim@student.ubc.ca (M.M.Y.); ritajyx@student.ubc.ca (Y.X.J.); 2 Department of Ophthalmology and Vision Sciences, University of Toronto, Toronto, ON M5S 2L9, Canadapyan@kensingtonhealth.org (P.Y.); 3 Department of Ophthalmology and Visual Sciences, The University of British Columbia, Vancouver, BC V5Z 3N9, Canada; haarism27@gmail.com (H.K.);

**Keywords:** diabetic retinopathy, circulating cell-free DNA, epigenetic, biomarker, personalized medicine, precision medicine, sequencing

## Abstract

Diabetic retinopathy (DR), a complication of type 2 diabetes mellitus (T2DM), is typically asymptomatic in its early stages. Diagnosis typically relies on routine fundoscopy for the clinical detection of microvascular abnormalities. However, permanent retinal damage may occur well before clinical signs are appreciable. In the early stages of DR, the retina undergoes distinct epigenetic changes, including DNA methylation and histone modifications. Recent evidence supports unique epigenetic ‘signatures’ in patients with DR compared to non-diabetic controls. These DNA ‘signature’ sequences may be specific to the retina and may circulate in peripheral blood in the form of cell-free DNA (cfDNA). In this review, we explore the literature and clinical application of cfDNA sampling as an early, non-invasive, accessible assessment tool for early DR detection. First, we summarize the known epigenetic signatures of DR. Next, we review current sequencing technologies used for cfDNA detection, such as magnetic bead-based enrichment, next-generation sequencing, and bisulfite sequencing. Finally, we outline the current research limitations and emerging areas of study which aim to improve the clinical utility of cfDNA for DR evaluation.

## 1. Introduction

### 1.1. Background on Diabetic Retinopathy

Diabetic retinopathy (DR) is a significant complication of diabetes mellitus, characterized by progressive damage to the retinal microvasculature. Globally, DR affects approximately 35% of diabetic patients, with nearly 10% progressing to stages of severe vision loss or blindness [1]. Among individuals with type 2 diabetes mellitus (T2DM), over 60% develop retinopathy after 20 years of living with the condition [2]. This progression is driven by pathological changes in the retinal vasculature, where chronic hyperglycemia leads to increased vascular permeability, tissue ischemia, and aberrant neovascularization, resulting in complications such as macular edema, vitreous hemorrhage, and retinal detachment [3].

### 1.2. Importance of Early Detection

DR is a multifactorial disease, presenting in 13% of newly diagnosed T2DM patients, and is typically asymptomatic in its early stages [3]. Initial detection relies on routine fundoscopy and clinical assessment of microvascular abnormalities [4]. However, during this asymptomatic phase, subclinical neural retinal damage and subtle microvascular changes begin to progress [5]. Early detection may encourage a more proactive lifestyle and pharmacological modifications to preserve retinal function [6]. 

Fundoscopy, a non-invasive clinical examination technique, is the current gold standard for DR diagnosis [7]. While a fundus examination is recommended annually for diabetic patients, it has significant limitations. It requires pupil dilation, detects only irreversible structural changes, and, in patients with mobility limitations, may present a restricted retinal view [8]. Electroretinography (ERG), an alternative technique for DR detection, functions by measuring the electrical responses of retinal cells to light stimulation [5,9,10,11,12]. However, ERG testing requires a certified practitioner and is associated with high costs [13]. Despite these limitations, ERG is a useful modality to monitor for early molecular changes. 

### 1.3. Emerging Role of Epigenetics

Epigenetics is a rapidly growing area of study that is often applied to treat complex, multifactorial diseases. Epigenetics involves DNA structural modifications that do not alter the underlying sequence, yet nonetheless influence gene expression and cellular phenotype [14]. Unlike genetic mutations, epigenetic modifications are reversible and can be influenced by cellular and environmental factors [14]. Profiling these epigenetic signatures using molecular techniques can outline the early presence and severity of metabolic disorders at a personalized level [15]. Due to the reversible nature of epigenetic changes, several small molecules, known as “epi-drugs”, are being developed to reverse gene alterations and promote more favourable outcomes; the first of these drugs has been approved for consumer use in cancer treatment [16,17]. Many studies are currently exploring this avenue for metabolic disorders [18,19,20].

**Figure 1:**
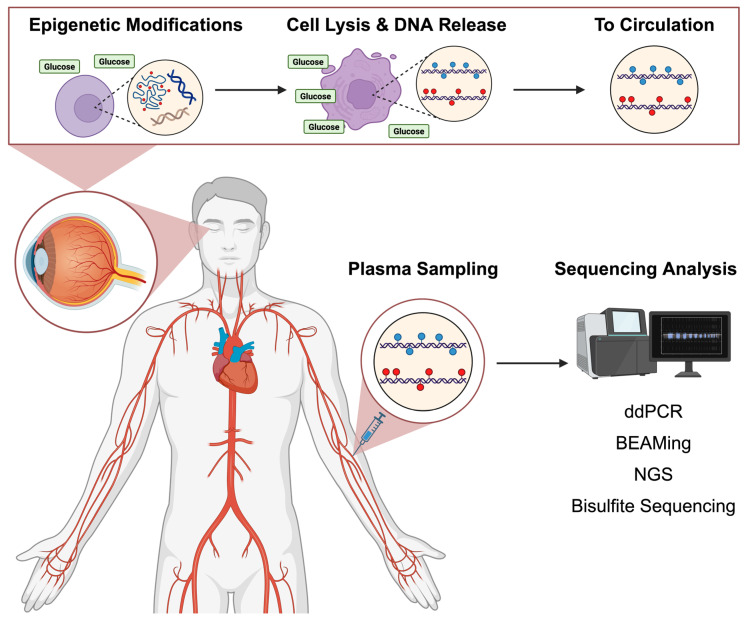
Visual summary of the origin, collection, and analysis of cfDNA in relation to DR. The diabetic milieu forms DR-specific epigenetic profiles and promotes cell lysis, releasing altered DNA into circulation as cfDNA, which is subsequently collected through plasma sampling. Sequencing methods such as digital droplet PCR (ddPCR), Magnetic Bead-Based Enrichment (BEAMing), next-generation sequencing (NGS), bisulfite sequencing then identify these distinct epigenetic profiles for early DR detection.

In DR, the retina undergoes distinct epigenetic alterations, which are detectable even in early or sub-clinical cases, suggesting they play a role in disease progression [21,22]. The diabetic environment induces a metabolic disturbance in circulating cells, which subsequently alters their gene expression patterns as a compensatory mechanism [21,22]. This process facilitates epigenetic modifications that ultimately drive cellular dysfunction (Figure 1) [23]. The current evidence supports an association between specific epigenetic profiles and microvascular complications in early DR, suggesting that epigenetic variations may precede structural damage [23]. Therefore, the efficient epigenetic sampling of patients may offer a promising approach to providing an early and personalized insight into patients’ risk of DR [24].

### 1.4. Circulating cfDNA as a Biomarker for DR

The diabetic milieu induces metabolic stress within the retinal endothelium and neuronal structures [25]. Chronic exposure to this stress leads to cell death, resulting in the release of free nuclear and mitochondrial DNA (mtDNA) into the local bloodstream, which thereafter circulates systemically [26]. Epigenetic modifications from DR in the retinal cells can be measured through pooling the circulating cell-free DNA (cfDNA) in the peripheral blood [26]. Current sequencing technologies are capable of accurately detecting pathology-signifying epigenetic features from cfDNA (Figure 1), and this technique is well-established clinically to detect genetic mutations in cancers [27,28,29,30,31].

While multiple epigenetic modifications have been linked to DR [15,32], the use of cfDNA-biomarkers for real-time DR monitoring remains underexplored. In this review, we will (1) identify unique epigenetic signatures in DR, (2) critically assess cfDNA testing as an early diagnostic tool for the condition, and (3) discuss directions for future improvements. Ultimately, we will evaluate the potential of cfDNA epigenetics for non-invasive, early DR detection and management.

## 2. Pathophysiology of DR and cfDNA Origin

### 2.1. Vascular and Neural Apoptosis Contributing to the Accumulation of cfDNA

Chronic hyperglycemia induces cell metabolic changes, which negatively impact the survival of retinal endothelial and neuronal cells [33,34]. These changes result in the production of reactive oxygen species (ROS) that elicit oxidative stress within the retinal tissues, since ROS molecules contain unpaired electrons that participate in redox reactions with cellular macromolecules [33,34]. Under normal conditions, the impact of cellular ROS is reduced by antioxidants, such as superoxide dismutase (SOD), which convert ROS to non-reactive oxygen and water products to prevent aberrant reactions [33,34]. In DR, increased circulating glucose concentrations facilitate a hypermetabolic state in cells, causing ROS overproduction and an oxidant–antioxidant imbalance. ROS accumulate over time, thereafter eliciting downstream metabolic pathways which ultimately result in oxidative stress and cell apoptosis [35].

Oxidative stress in retinal cells results in the accumulation of advanced glycation end-products (AGEs), which ultimately promotes a hypermetabolic state [36]. AGEs induce thickening and extracellular matrix changes within the retinal capillary basement membrane [36]. They enhance capillary stiffness and limit growth factor permeability across the endothelial membrane, ultimately leading to apoptosis of both pericytes and endothelial cells [25,37]. Both human and animal studies suggest that hyperglycemia triggers pericyte apoptosis [38,39]. Pericytes normally provide structural support to retinal capillaries, and their loss impairs vessel integrity and further causes blood supply impairment toward the neuroretina [40].

Ganglion cells and the supportive Müller glial cells constitute important components of the retina, which carries visual signals to the brain [41]. Multiple in vitro studies demonstrate an association between DR and neurodegeneration. First, animal studies support that apoptosis of retinal neurons may be an early event during diabetes induction [42]. Second, in diabetic animals and subjects, the upregulation of pro-apoptotic molecules such as cleaved caspase-3, Bax, and Fas has been observed in retinal neurons [43,44,45]. Lastly, mitochondrial dysfunction has been associated with neurodegeneration in DR. In the donor eyes of diabetic individuals, the retinal expression of pro-apoptotic mitochondrial proteins such as cytochrome c and apoptosis-inducing proteins was found to be significantly elevated [44].

In summary, DR occurs secondary to hyperglycemia-induced retinal vascular damage related to underlying capillary endothelial breakdown from cell apoptosis [33,34]. DNA released from neural and vascular apoptotic cells are degraded by intracellular nucleases into fragments, which are then released into the bloodstream [46]. Circulating cfDNA may be quite limited in healthy individuals, as it constitutes less than 10% of total cellular DNA and has a relatively short circulating half-life, ranging between 16 minutes and 2.5 hours, due to rapid degradation by local nucleases [46,47,48,49]. In DR, there is a greater rate of cell apoptosis, especially among retinal pericytes, endothelial cells, and retinal ganglion cells, all of which contribute to the cfDNA pool [50]. Indeed, cfDNA levels have been reported to be elevated in patients with DR, likely resulting from the apoptosis of these various cell types [51,52].

### 2.2. DR Epigenetic Dysregulation

Both genetic predisposition and environmental factors play an important role in diabetes risk and progression [53,54,55]. Epigenetics involves non-permanent DNA modifications influenced by both the host and cell environment [14]. These changes alter the gene expression profile, which can, in turn, further shape the cell environment [14]. Two primary types of epigenetic changes seen in DR are the DNA methylation and histone modifications induced by oxidative stress [56]. 

Nuclear and mtDNA are methylated by DNA methyltransferase in response to pathological and environmental factors, whereas S-adenosylmethionine functions as a methyl group donor [57]. Methylation impedes DNA binding to other transcription factors and generally acts to silence gene expression [57]. DNMTs primarily exert their enzymatic activity on the cytosine base of CpG dinucleotides. As a result, methylation modifications predominantly occur in CpG-rich sequences, known as CpG islands [57]. These regions are typically found in gene-promoter regions and heavily alter gene expression levels [57]. Hyperglycemia-induced oxidative stress will modify the epigenetic features of DNA and initiate a positive feedback loop eliciting further disease pathogenesis [58,59]. Human studies also show elevated global DNA methylation during early disease onset specific to T2DM patients with DR, suggesting that measuring DNA methylation would be a useful, non-invasive, biomarker to indicate early changes in DR [60].

DNA is wrapped around nucleosomes, each consisting of two histone proteins. Epigenetic modifications of these histones regulate the ‘tightness’ of DNA wrapping, influencing transcription factor-binding and, ultimately, gene expression [21]. Histone epigenetics involve either the methylation or acetylation of certain histone amino acids according to their receptive enzymes. Histone methylation of some positively charged amino acids may disrupt ionic interactions between the positively charged histone and negatively charged DNA, and may be associated with gene modulation [61].

## 3. Epigenetic Signatures in DR

### 3.1. DNA Methylation Signatures

Methylation changes form a complex network of interactions leading to disease pathogenesis, and while the exact mechanism is unknown, many studies associate certain epigenetic modifications with DR [60,62,63]. In a case–control study, Maghbooli et al. reported an elevation in global DNA methylation during the primary disease onset of DR, relative to non-DR T2DM controls. Further, they found an increase in DNA methylation with DR progression, suggesting that DNA methylation is not only suggestive of DR but may also correlate with its severity (Table 1) [60,62]. In support of this theory, DNMT activity is elevated in the retina of diabetic patients, suggesting DNA hypermethylation activity [64]. Methylation effects on specific promoters have also been linked to DR risk and progression. For instance, 5,10-methylenetetrahydrofolate reductase (MTHFR) is an enzyme involved in the methionine–homocysteine cycle [62,65,66]. Hypermethylation of the *MTHFR* promoter is significantly associated with DR, as well as elevated total cholesterol, LDL cholesterol, and glucose levels [62,65,66]. Further, studies report that 5-hydroxymethylcytosine (5hmC), a methylated form of DNA base cytosine in cfDNA, plays a role in gene regulation [67]. In patients with DR, 5hmC was associated with regions of histone modification, such as histone H3 lysine4 monomethylation (H3K4me1) (Table 1) [32]. This colocalization indicates a regulatory role of 5hmC in the gene expression of DR populations. Moreover, the researchers also reported a three-gene signature associated with DR (*MESP1*, *LY6G6D*, *LINC01556*), thereby supporting the idea that the expression level of certain genes may be relevant as a surrogate marker of DR (Table 1) [32]. Many studies have shown that global DNA methylation and the hypermethylation of specific gene-promoters are associated with an increased risk of DR [32,60,65,66,68,69]. Future studies are beginning to investigate the epigenetic changes in mtDNA related to the pathogenesis of DR [21]. Despite accounting for less than 1% of total cellular DNA, mtDNA contains approximately 450 CpG sites and 4500 cytosines at non-CpG sites [70]. While mtDNA methylation is strongly linked to various chronic diseases, such as cancer, its role in metabolic diseases remains relatively underexplored [21].

### 3.2. Histone Modification Signatures and Changes to Gene Expression

Histone modifications are associated with the prevention of antioxidant production and the increased expression of apoptotic pathway markers [23,39]. Studies show an increase in histone deacetylase and decreased histone acetylase activity in DR models, suggesting alterations in histone acetylation with disease. Indeed, their activity in DR contributes to increased oxidative stress [71]. In cells, ROS is normally balanced by antioxidants, primarily SOD, to maintain oxidative balance [33,34]. However, in the pathogenesis of DR, hyperglycemia increases the binding of a co-repressor, lysine-specific demethylase 1, which demethylates H3K4me1 and H3K4me2 (Table 1) [23,72]. This leads to transcriptional repression of the MnSOD-encoding gene, *Sod2*, effectively reducing antioxidant production and further increasing ROS-related DR pathogenesis [23,72].

Overall, the distinct DNA methylation and histone modification profiles found in DR suggest that epigenetic signatures can be used to detect DR, along with a potential therapeutic target to halt its progression. 

**Table 1:** Epigenetic signatures for DR (partly adapted from Milluzzo et al., 2021) [62].

Author and Year	Origin	Sample	Marker	T2D Groups (n)	Control Group (n)	Main Results
Han et al., 2021 [32]	China	Plasma	*MESP1*, *LY6G6D*, and *LINC01556*	DR (35)	Age-, gender-, and diabetic duration-matched T2DM (35)	Three-gene signature active expression associated with DR.
Maghbooli et al., 2015 [60]	Iran	Peripheral blood leukocytes	Global DNA methylation (5-methylcytosine content)	PDR, NPDR (74), NDR (94)	None	Increased global DNA methylation associated with PDR, NPDR, and NDR.
Nunes et al., 2017 [65]	Brazil	Peripheral blood leukocytes	Methylation of *MTHFR*-promoter and polymorphism of 1298AA of *MTHFR*	DR (16), DN (29)	T2DM with no complications (60)	*MTHFR*-promoter hypermethylation associated with DR.
Bezerra et al., 2019 [66]	Brazil	Peripheral blood leukocytes	Methylation of *MTHFR*-promoter and polymorphism of C677T and A1298C of *MTHFR*	DR (22), NDR (25)	T2DM with no complications (60)	*MTHFR*-promoter hypermethylation with 1298AA polymorphism is associated with higher glycemia, LDL cholesterol, and total cholesterol.
Nunes et al., 2018 [68]	Brazil	Peripheral blood leukocytes	Methylation of *miR-9-3-*, *miR-34a-*, and *miR-137*-promoters	DR (19), DN (29)	T2DM with no complications (60)	*miR-9-3*-promoter hypermethylation associated with increased risk of DR. *miR-137*-promoter hypermethylation associated with protective effects, reducing microvascular diabetes complications.
Duraisamy et al., 2019 [69]	USA	Blood	*MHL1* and *SOD2* hypermethylation	PDR (23)	Non-DR-T2DM (23); healthy control (15).	Higher 5mC levels in the *SOD2*-promoter resulted in a 50% decrease in *SOD2* mRNA in PDR and a 20% decrease in non-DR-T2DM.
Yang et al., 2022 [73]	China	Blood	*ZDHHC23* and *SLC25A21* hypermethylation	T2DM with DR (43)	T2DM without DR (92)	Hypermethylation of cg12869254 and cg04026387, containing the *ZDHHC23* and *SLC25A21* genes
Han et al., 2021 [32]	China	Blood	H3K4me1	DR (35)	Age-, gender-, and diabetic duration-matched T2DM (35)	H3K4me1 active expression associated with DR.

Abbreviations: PDR = proliferative DR; NPDR = non-proliferative DR; DR = diabetic retinopathy; NDR = not DR; DN = diabetic nephropathy; T2DM = type 2 diabetes mellitus.

## 4. CfDNA in Detection Technologies

### 4.1. Origin of cfDNA in Circulation

Apoptosis is widely recognized as the predominant mechanism of retinal cell death, with minimal evidence supporting necrosis as a significant contributor during early disease stages [74]. Hyperglycemia-induced oxidative stress in retinal cells triggers Bax/Bcl-2 dysregulation, leading to caspase-3 activation, DNA fragmentation, and subsequent pericyte dropout and capillary basement membrane-thickening [43,44,45]. This cascade enhances caspase-activated DNase (CAD) activity, which cleaves chromatin at the linker regions between nucleosomes, producing DNA fragments of approximately 167 base pairs [75]. A fragmentation pattern of 167 base pairs is characteristic of nucleosome-protected DNA, since nucleosomes wrap approximately 147 base pairs of DNA with an additional 80 base pairs that are contributed by linker regions [45,73,75]. While this fragmentation pattern is not unique to apoptosis, apoptotic cleavage by CAD preferentially occurs in linker regions, reinforcing the predominance of nucleosome-associated cfDNA fragments in apoptosis-driven conditions such as DR [76,77]. TUNEL assays support this pattern of apoptotic cfDNA fragmentation in diabetic retinas, as opposed to non-diabetic controls, which do not display such a pattern [74,77]. Consequently, the cfDNA fragmentation patterns contains epigenetic modifications that offer diagnostic utility in mapping the patient’s epigenetic profile to their disease [74,77].

These cfDNA fragments preserve the epigenetic modifications of their origin cells, including DNA methylation (5-methylcytosine, 5mC) and hydroxymethylation (5hmC) [32]. CfDNA methylation and hydroxymethylation are chemically stable modifications, allowing cfDNA to serve as a less-invasive biomarker that reflects the epigenetic landscape of its origin tissue [78,79]. High-throughput analyses have demonstrated that cfDNA methylation profiles closely resemble those of the originating tissue [80]. For instance, Shinjo et al. report that over 80% of the methylated regions identified in pancreatic tumours were also present in patient plasma cfDNA with a strong correlation, indicating that cfDNA reflects the DNA methylation landscape of its source cells [80]. The genome-wide 5hmC profiling in DR patients revealed enrichment at active chromatin sites in retinal cells, aligning with histone marks associated with gene activation, such as H3K4me1 [32]. Although standard cfDNA isolation primarily recovers DNA fragments (with histones dissociating during extraction), fragmentation patterns still reflect chromatin structure [32]. In one study, 5hmC regions were detected in the cfDNA from DR patients co-localized with active regulatory regions, suggesting that cfDNA is capable of mapping key regulatory regions that elicit cell phenotype changes [32].

### 4.2. Tissue-Specific cfDNA from Retinal Cells

Circulating cfDNA retains the epigenetic hallmarks of its tissue of origin, including DNA methylation, hydroxymethylation, and nucleosome positioning patterns [32]. CfDNA methylation analysis enables the identification of retinal-derived DNA fragments, as many genes exhibit tissue-specific methylation patterns [80,81]. Genes such as *RHO*-encoding rhodopsin and *RBP3*-encoding interphotoreceptor retinoid-binding protein show retina-specific hypomethylation, and consequently can distinguish retinal cfDNA from other tissue-derived fragments [81]. Additionally, the developmental transcription factors *PRDM13* and *ATOH7*, involved in retinal development, exhibit unique methylation patterns that further support a retinal cfDNA origin [82]. The unmethylated sequences detected in these genes in plasma provide further evidence of retinal cell apoptosis and could serve as an early biomarker for DR-related retinal damage.

Beyond methylation analysis, nucleosome footprinting further supports tissue identification through analyzing how cfDNA fragmentation patterns correspond to chromatin accessibility [77]. As nucleosomes protect DNA from degradation, cfDNA fragments are preferentially cleaved by DNases in nucleosome-free regions [77]. Different tissues exhibit a unique nucleosome positioning, which influences the fragmentation profiles of cfDNA [77]. In retinal cells, genes with open chromatin tend to have distinct cfDNA fragmentation patterns, often showing periodic peaks around transcription start sites [83]. These fragmentation signatures can be detected in plasma samples and serve as a feature to identify retinal-specific cfDNA with various detection and quantification techniques [83].

### 4.3. Detection and Quantification Techniques

#### 4.3.1. DdPCR

DdPCR is a highly sensitive method for targeted cfDNA quantification, utilizing nanodroplet partitioning to amplify DNA through PCR [27,83]. It detects low-frequency DNA fragments (~0.01%) with minimal input (~5–10 ng) and offers rapid processing times [27,83]. DdPCR is suitable for monitoring known epigenetic biomarkers, such as unmethylated or methylated fractions of tissue-specific genes [27,83]. However, it is limited to predefined targets and does not facilitate novel biomarker discovery (Table 2) [27,84].

#### 4.3.2. BEAMing

BEAMing (beads, emulsification, amplification, and magnetics) is a highly sensitive digital PCR-based technique that combines flow cytometry and magnetic bead separation to enable the detection of rare cfDNA fragments [49]. BEAMing achieves sensitivity levels as low as 0.01% for the mutant allele fraction, making it advantageous for early-stage disease detection, including the detection of epigenetic alterations in cfDNA [85,86]. Unlike conventional PCR, BEAMing allows for a high-throughput analysis with single-molecule sensitivity and has been effectively utilized in cancer diagnostics and liquid biopsies (Table 2) [85,86].

#### 4.3.3. NGS

NGS enables comprehensive epigenetic analysis through targeted panels or genome-wide sequencing [87]. Targeted panels focus on predefined CpG sites, while genome-wide approaches, such as cell-free methylated DNA immunoprecipitation and high-throughput sequencing, facilitate the identification of novel methylation patterns [87]. Although NGS requires an increased DNA input (~20–100 ng) and longer processing times, it provides a detailed epigenetic landscape, including nucleosome positioning and 5hmC distributions [27,80,87,88]. This versatility makes it a powerful tool for both the discovery and validation of epigenetic biomarkers [86,89]. A specialized NGS technique, Tagged-Amplicon Deep Sequencing (TAm-Seq), enhances the detection of low-abundance cfDNA variants by employing unique molecular identifiers for the precise quantification of rare methylation changes [86,89]. Compared to conventional NGS methods, it requires a lower DNA input while maintaining high analytical sensitivity, which is advantageous for tracking disease progression and identifying early epigenetic biomarkers in cfDNA (Table 2) [86,89].

#### 4.3.4. Bisulfite Sequencing

As the gold standard for detecting DNA methylation at a single-base resolution, bisulfite sequencing converts unmethylated cytosines to uracils, enabling the precise quantification of methylation levels [79]. Despite the DNA degradation that occurs during conversion, it offers high specificity and accuracy [79]. Bisulfite sequencing is crucial for both targeted analyses and genome-wide methylome studies, facilitating the identification of differentially methylated regions relevant to tissue-specific epigenetic profiling (Table 2) [27,88,90].

**Table 2:** Comparison of current cfDNA sequencing technologies for epigenetic signatures.

Analysis	Type	Sensitivity	Target	Advantages	Limitations	First Author and Year
DdPCR	Targeted	~0.01%	Known epigenetic markers	High sensitivity; low DNA input; fast results	Limited to known targets, limited multiplex ability	Medina et al., 2023; Hindson et al., 2013 [27,84]
BEAMing	Targeted (Digital PCR)	~0.01%	Rare epigenetic variants	High accuracy; detects ultra-low variant levels	Requires specialized equipment	Diehl et al., 2008; Pircalabioru et al., 2024 [85,86]
NGS (Targeted)	Targeted	Moderate	Specific CpG sites	Broad analysis; scalable	Requires assay design; longer processing time	Medina et al., 2023; Shinjo et al., 2020; Koval et al., 2021 [27,80,88]
NGS (Genome-wide)	Unbiased	High	Entire methylome	Discovery of novel markers	High DNA input; resource-intensive	Medina et al., 2023; Shen et al., 2019 [27,87]
Bisulfite Sequencing	Targeted or genome-wide	High	Single-CpG resolution	Quantitative and specific	DNA degradation; cannot distinguish 5mC from 5hmC	Medina et al., 2023; Koval et al., 2021; Zhang et al., 2015 [27,88,90]

## 5. Current Limitations and Future Directions

### 5.1. Limitations in cfDNA Sequencing

CfDNA can be evaluated via various methods, such as ddPCR, beads, emulsion, amplification, and BEAMing, TAm-Seq, and whole-genome bisulfite sequencing [91]. The limitations of analyzing cfDNA as a biomarker for early DR diagnosis include limitations involving each method’s detection sensitivity and requirements. Highly sensitive tools like ddPCR have been previously used to quantify cfDNA from urine samples in combination with techniques like NGS or targeted amplification methods like long terminal repeat amplification [92]. However, while ddPCR is highly sensitive and capable of quantifying cfDNA markers as low as 0.01–1.0%, it is not sufficient on its own to diagnose DR [86]. Firstly, ddPCR provides an absolute quantification of cfDNA markers, which can be influenced by non-disease factors such as age, inflammation, and other comorbidities [93,94]. Secondly, ddPCR relies on sequence amplification with primers and probes, which are only designed for well-characterized sequences and can only evaluate one genomic aberration at a time [92,95]. This poses a limitation in studying multiple, novel cfDNA markers to further reliable DR diagnosis. Furthermore, the selected primers and probes can exhibit non-specific binding to non-DR-specific cfDNA markers, causing biases in analyses [96].

BEAMing is a highly sensitive and cost-effective method to study cfDNA changes that rely on known mutations [97,98,99]. Similarly, the TAm-Seq method offers high specificity (97% accuracy), sensitivity (detection level of 2%), and throughput [100,101,102]. While cfDNA markers can be useful in the absence of structural changes, allowing for early DR diagnosis, some sequencing modalities for analyzing cfDNA, such as BEAMing and TAm-Seq, require well-characterized sequences [95]. This raises the concern that more research needs to be conducted for the large-scale validation of these sequences and their generalizability across diverse patient populations.

The use of cfDNA markers in DR diagnosis is delicate, as both oversaturation and the excessive degradation of these markers can lead to false results. Oversaturation occurs when cfDNA levels exceed the detection range of the analytical method. This can result from comorbidities, inadequate sample dilution, and improper assay choice [93,94]. This is mitigated by sample dilution, assay optimization, and multiplexing to improve precision [103,104]. In contrast, the excessive degradation of cfDNA can bias sequencing analysis. Upon release from cells, cfDNA is degraded by enzymes such as DNases into smaller fragments, resulting in fragment size bias, where shorter fragments are favoured for sequencing. Studies show that the fragmentation pattern of cfDNA is associated with its epigenetic profile [77,105]. Thus, fragmentation bias can result in the overrepresentation of shorter cfDNA fragment markers in disease states and interfere with the reliability of the cfDNA fragment size profiles. Low levels of cfDNAs (<1% of reads) can further face difficulties in distinguishing positive signals from background noise [106]. 

### 5.2. Epigenetic Variability and the Future of cfDNA in Precision Medicine

An individual’s epigenetic profile can vary based on demographic variations and environmental exposures [107,108,109,110]. For instance, DNA methylation levels differ at the cytosine–phosphate–guanine sites between African American and Caucasian subjects [108]. Previous research established common epigenetic signatures characteristic of DR (see Table 1). However, these studies were often conducted in a single medical centre, suggesting the inclusion of an isolated racial group subset. Future studies can incorporate multi-centred collaborations to investigate the common epigenetic profiles of various demographics using a standardized protocol. Nevertheless, cfDNA holds promising potential for clinical implementation in the future of pharmacogenetics and precision medicine [111].

## 6. Conclusions

DR is a multifactorial disease that affects at least 60% of T2DM individuals and often presents with no patient-reported symptoms during early onset [2]. Initial detection using routine fundoscopy for clinically apparent microvascular damage is often possible only after the occurrence of subclinical irreversible retinal damage. The analysis of epigenetic profiles in cfDNA offers potential for early disease detection and monitoring, enabling diagnosis before clinical symptoms manifest and improving patient outcomes [4,5,112]. An analysis of DNA epigenetic profiles in patients using cfDNA offers an early, non-invasive diagnostic option. Current studies have identified epigenetic biomarkers specific to DR, and when paired with modern sequencing technologies, they enable the detection of these epigenetic changes in cfDNA [62]. While there is still room for improvement in their sensitivity and the development of comprehensive epigenetic databases, cfDNA offers a promising approach for future DR diagnosis and lays the foundation for patient-specific clinical evaluative strategies.

## Data Availability

No data were created in the production of this work.

## Abbreviations

The following abbreviations are used in this manuscript:

DR	Diabetic retinopathy
T2DM	Type 2 diabetes mellitus
cfDNA	Circulating cell-free DNA
ERG	Electroretinography
mtDNA	Mitochondrial DNA
ROS	Reactive oxygen species
SOD	Superoxide dismutase
AGE	Advanced glycation end-product
MTHFR	Methylenetetrahydrofolate reductase
5hmC	5-hydroxymethylcytosine
CAD	Caspase-activated DNase
ddPCR	Digital droplet PCR
BEAMing	Magnetic bead-based enrichment
NGS	Next-generation sequencing
TAm-Seq	Tagged-amplicon deep sequencing
PDR	Proliferative diabetic retinopathy
NPDR	Non-proliferative diabetic retinopathy
NDR	Not diabetic retinopathy
DN	Diabetic nephropathy
